# Same same but different: why we should care about the distinction between professionalism and ethics

**DOI:** 10.1186/s12910-016-0128-y

**Published:** 2016-07-22

**Authors:** Sabine Salloch

**Affiliations:** Institute for Ethics and History of Medicine, University Medicine Greifswald, Ellernholzstr. 1-2, D-17487 Greifswald, Germany

**Keywords:** Professionalism, Ethos, Ethics, Sociology of the Professions, Professional Law, Physician-Assisted Suicide

## Abstract

Medical professionalism forms a belief system which is used to defend physicians’ ethos against counterforces which might threaten the integrity of medical practice. The current debates on professionalism, however, are characterized by the lack of a clear distinction between professional and ethical aspects of physicians’ conduct. This article argues that a differentiation between professionalism and ethics is not of mere academic interest. Instead, it is of great practical importance with regard to morally contentious issues in medicine.

A short analysis of the discussions in history and social sciences reveals that professionalism is more than a catchphrase of modern medical debates but has a complex theoretical background which is still not conclusively understood. Whereas professionalism is clearly linked to the honorable aims of providing services to the individual and the society, it potentially entails problematic aspects, such as elitism, monopoly or the maintaining of power and privileges. With regard to morally contentious topics, the professional ethos of physicians must be differentiated from the perspective of ethics which can take a universal standpoint and has the potential to critically assess context-specific moral norms. The example of the current regulation on suicide assistance in German professional law is taken as an example to demonstrate how professional bodies tend to overstep the limits of their expertise and regulatory power with regard to issues which need an ethical evaluation.

The article concludes that the narrowing of ethics and professionalism in public discussions and in medical education should be seen as problematic and that morally contentious topics in modern societies should be open to a participatory and inclusive discussion and democratic decision procedures.

## Background

Especially since the launching of the Charter on Medical Professionalism in 2002 [1] the claim for a renewed sense of professionalism has been raised in divergent national contexts and healthcare systems [2–5]. Stressing professional autonomy and integrity is deployed as a strategy to oppose factors, such as emerging technologies, changing market forces or globalization, which might threaten the traditional role and privileges of physicians. The professional as a person is, thereby, often depicted in a rather idealistic manner showing “everything that we admire in our colleagues and strive for in ourselves” ([6], p. 1532). Following this image, the professional stands as a solid rock in the center of changing societies and healthcare systems while keeping up with the commitment to serve both the benefit of patients and the welfare of society [7].

In dealing with the topic of professionalism we should be aware of the fact that professionalism has two sides: it forms an important subject of historical and sociological sciences, but, at the same time, is an ideology or belief system in the medical community itself which is used to defend the inherent values of physicians’ ethos. Currently, the reflections on professionalism as a scientific concept and its theoretical embedding often remain unconsidered in the more practice-oriented societal and political discussions. The large historical and socio-theoretical contexts of referring to physicians as professionals are often not sufficiently recognized. A slightly deeper analysis, however, leads us to questions such as: What is the characterizing distinction between a profession and a mere job or business? What is the specific role of medical professionals in (post)modern societies? Are there normative claims which can be derived from the fact of being a professional?

The practical debates on professionalism are further blurred by the lack of a clear distinction between “professional” and “ethical” aspects in healthcare. Both sides are typically voiced in one breath when referring to physicians’ “ethical and professional values.” [8, 9] We are confronted with a large number of historical and contemporary codes which express the “professional and ethical duties” of physicians, nurses and other healthcare staff [7, 8, 10], but do not clearly differentiate between both sides. The line between professionalism and ethics is also not clearly drawn in medical education. Training programs and Centers for Ethics and Professionalism aim to ensure healthcare professionals’ orientation towards values and provide them with the respective knowledge, skills and attitude [11, 12]. Newly qualified physicians in some countries are obliged to demonstrate and revalidate their professional development in predefined intervals [13].

There are several attempts to systematically explain the basis of physicians’ professional ethos. Most often it is related to a so-called “internal morality” of medical practice and the inherent goals of medicine such as preventing and curing, relieving pain and disability or helping the patient to live with his disease. Professional ethos is then linked with a virtue-ethical account which highlights character traits indispensable for an attainment of the ends of medicine ([14], p. 381). Under these premises professional organizations and associations are extensions of the ethical and moral commitments which are shared by the physician community ([14], p. 382). Whereas major physician duties remain rather unquestioned from the viewpoint of medicine’s internal morality there are also “borderline activities” which occupy a controversial status ([15], p. 390 ff.). Physician-assisted suicide (PAS) forms an example of such a practice where the traditional morality of medicine has to be critically re-evaluated to examine whether assisted suicide in certain circumstances is permissible ([15], p. 397). For an evaluation of morally contentious practices in medicine a reference to physicians’ professional virtues might often not be sufficient but an ethical evaluation is needed which oversteps the focus on the inherent aims of medicine and broadens the scope to a more general estimation of the phenomena which are under consideration from an ethical viewpoint. In the case of PAS, for example, the fact that medicine is supposed to help the patient to die with dignity and peace ([15], p. 387) does not suffice to answer the question whether physicians can assist the patient to end her life deliberately under defined circumstances.

A clearer distinction between the perspectives of ethics and professionalism with regard to medical borderline cases is therefore necessary. Whereas a number of duties, such as the commitment to patient autonomy, public accountability or scientific excellence, can be framed from the perspective of professionalism and ethics likewise, the difference between both is far from being of mere academic interest. Instead, there is the need for a reflection on the limits of professionalism as a group-specific moral orientation. In this paper, I provide some necessary conceptual clarifications on the notion of medical professionalism and its historical and socio-theoretical embedding. The focus is then laid on professional organizations’ right to self-regulation, which has been transferred to them by the state, but is restricted to those issues which form part of the members’ professional expertise. Problems which arise from overstepping the boundaries of professional self-regulation are highlighted using the example of the position towards PAS in German professional law. I will argue that the question whether physicians should be allowed to assist patients in ending their life deliberately should not be a subject of professional self-regulation. Instead, the legitimacy of suicide forms part of a comprehensive ethical debate and its contemporary interpretation should be left to democratic decision procedures.

## Discussion

### Professionalism – historical and sociological perspectives

Although professionalism today forms a highly prominent notion, especially in medical education, the complex and multi-branched discussion on professionalism in the historical and social sciences is often not recognized. The predominant general evaluation of the professions in the scientific literature is a positive one and stresses the professionals’ orientation towards serving the needs of individuals and the public. However, there are also problematic aspects, such as the monopolistic character of the professionalized market or privileged private governments run by the professions [16]. From a historical perspective, the rise of professionalism started with the medieval guilds, which became the organizing principle for skilled work in many European cities. Guilds controlled the handwork with regard to aspects such as the quality of the goods or the rules for apprenticeship and for advancement to a master craftsman status. In this, the guilds maintained a public good (the permanent and reliable provision of products), but, at the same time, created comfortable working conditions and a good income for their members ([6], p. 1533). Lawyers and doctors were among the first who began to form guilds in the late medieval and early Renaissance periods. Because of their university education, both groups belonged to the social elite. The further historical development of the guilds was then very much dependent on the political circumstances and on the rise of capitalism as a counterforce to the traditional guild system. Due to the divergent evolution of the economic systems, main differences can be observed in the development of professionalism between various European countries and the US [6, 17].

Professional organizations which replaced the guilds in modern times were still powerful at regulating highly important social goods, such as jurisdiction or health. However, they were much more dependent on state regulations than the guilds, which again led to divergent developments in different countries and states. The professionalization of German physicians, for example, was longtime hindered by the various principalities, dependence on the wealthy and the lack of reliable medical knowledge. Under these circumstances, German physicians in the 19th century “were inclined to emphasize their membership of the ‘educated middle-class’ rather than their distinctive professionalism” ([17], p. 93). After unification and the building of the *Kaiserreich* in 1871, the German state was still in a very strong position and maintained a wide control over medicine and medical education ([17], p. 93). During the 20th century, German physicians’ associations then gained more and more power and were endowed by the state with important rights to self-regulation. Today, the federal character of the medical professional system in Germany is maintained by the division of 17 Federal Chambers of Physicians (*Landesärztekammern*), which each have the regulatory power of professional jurisdiction over their members in their respective territory. They are entitled to adjudicate on the basis of their professional law and can inflict sanctions, such as admonition, rebuke or monetary penalty. Another professional privilege which has survived until today is the German physicians’ right to opt out of the state pension insurance and to create their own pension fund for above-average wealthy members.

The character of the professions as one exceptional type of occupation is not only of interest for historians, but has also been a decades-old topic in sociology. A main influence on the debate originates from Emile Durkheim, who depicted professions as entities that embody all valuable social forces in one. Subsequent to Durkheim, professions were the most stable elements in society for a long time which preserved and passed on traditions and functioned as “centres of resistance to crude forces which threaten steady and peaceful evolution” ([18], p. 497). In contrast to these functional approaches to the sociology of the professions, the school of Symbolic Interactionism in the mid-20th century developed an alternative view by referring to studies which investigated the interactions of individuals and social groups [19, 20]. These studies allowed for a more critical look at the actual day-to-day world of professionals and revealed ideologies and myths associated with the notion of professionalism. The so-called “power approach” to professionalism strove to explain how the medical profession gained autonomy and developed dominance over other occupations in various, mainly Anglo-American, contexts ([17], p. 4 f.). Proponents of the “power approach” typically stress professionals’ monopoly of distinctive services and are characterized, for example, by a critical attitude towards the alleged public welfare orientation of professions [21, 22].

Today the spectrum of approaches to the sociology of the professions is manifold. One of the most influential approaches was developed by Eliot Freidson, who argues that the internal structure of professionalism is neither congruent with the consumer-led free market nor is it comparable with a bureaucratic system of planned and controlled economy ([23], p. 1). Instead, professionalism follows a “third logic,” in which workers with a specialized knowledge have the power to organize and control their work. Following this ideal type of professionalism, experts are doing good work for their own satisfaction and, consequently, serve the needs of both consumers and managers. Freidson argues that monopoly and the notion of self-government are essential for professionalism: “In the most elementary sense, professionalism is a set of institutions which permit the members of an occupation to make a living while controlling their own work” ([23], p. 17). Freidson defends professionalism against various criticisms, such as the reproach of elitism and misguided privileges ([23], p. 206 ff.). His approach, thus, provides a fruitful basis for understanding the different manifestations of professionalism under a common organizing idea.

The brief overview of history and sociological theory reveals that the notion of professionalism is more than a catchphrase of modern medical debates, and has a complex theoretical background which is still not conclusively understood. It further shows that while, on the one hand, professionalism is linked to the honorable aims of providing services to the individual and the society, on the other hand, it entails potentially problematic aspects, such as elitism, monopoly or the maintaining of power and privileges. An uncritical equation (or at least narrowing) of professionalism and ethics in modern discussions is, therefore, highly problematic. The prerogative of self-regulation which distinguishes professions from other occupations comes along with the responsibility to respect the limits of the professions’ expertise with regard to issues which need an ethical evaluation which goes beyond a group-specific internal morality. In the next section, I will go further into the analysis of the relation between both realms, professionalism and ethics, with a special focus on the scope of professional self-regulation.

### Professional self-regulation and ethics

According to Eliot Freidson, the freedom of judgement or discretion in performing work is essential to professionalism ([23], p. 3). The “intellectual specialization” as a specific feature of professionals ([23], p. 21 ff.) can only be successfully exercised when professionals possess a sufficient degree of autonomy which allows them to make independent and self-reliant judgments in those issues which form part of their expertise. Institutions of professionalism play an important role by maintaining high-quality services. They are grounded in the “social enterprise of learning, advancing, and practicing a body of specialized knowledge and skill” ([23], p. 198). Professional organizations control training, certification and practice and are supposed to advance the refinement of knowledge and skill.

The exclusive knowledge and specialization of physicians creates the basis for professional organizations’ right to self-regulation. The community confers a series of powers and privileges upon the profession which enable it to exercise professional judgement for the best sake of their clients and society ([24], p. 48 ff.). In the case of physicians, this means that key aspects of exercising the medical job lie in the hands of self-legislating boards and committees. Depending on the respective national context, professional organizations govern the medical curriculum, the admission into the profession, the structure of the advanced training for specialization, the duties towards patients and colleagues, the permissibility of advertisement and much more. Many of these issues can effectively be best decided by experts who have the appropriate scientific knowledge, practical skill and experience. The idea of professional self-regulation, however, reaches a limit in those cases which not only go beyond the scope of medical expertise, but touch on value-laden existential questions, such as abortion, preimplantation genetic diagnosis, euthanasia or PAS. The intrusion of the scientific model of medicine into other areas has been highlighted with regard to normatively laden topics such as the definition of illness or the brain death discussion [25]. A medicalisation, i.e. the increasing treatment of a wide range of human conditions and problems as medical conditions, takes place with respect to various forms of deviant behavior and lets the “medical logic” become dominant in areas which have been considered as questions of general lifestyle or education before. On the other hand the emergence of bioethics in the last decades of the 20th century prompted a tendency to let medical “outsiders” (such as philosophers or social scientists) play a major role in discussing and regulating issues that used to be left to physicians [26].

The expertise which is needed to fully evaluate empirically complex moral topics in modern medicine and healthcare encompasses both, procedural and methodological aspects which are possessed by philosophers and other scientists as well as medical knowledge and experience contributed by the members of the medical profession. However, professional organizations have a tendency to independently regulate on topics which do not form a subject of medical expertise alone. As Eric Vogelstein convincingly argues there are two lines of argument by which professional organizations could claim the ethical authority on morally controversial topics which both finally fail [27]. According to the “argument from ethical discovery,” professionals have “special abilities or knowledge that would allow them to compose moral arguments or otherwise arrive at moral conclusions that are especially reliable” ([27], p. 5). However, the special knowledge which medical professionals have at their command does not particularly extend to those skills and knowledge which are important for *moral* reasoning. According to the second option, the “argument from ethical constructivism,” professionals “determine their professional ethical norms via agreement about the proper societal role, goals, boundaries, and standards of that profession” ([27], p. 6) However, Vogelstein argues that these norms must be subject to good moral reasons which can be discovered, but not created by the professionals: “There must be an ethical basis for determining any particular professional ethic – otherwise that basis will be arbitrary or capricious, reflective merely of the will of the relevant professionals” ([27], p. 6).

Vogelstein’s last argument can also be reframed as the opposition between professional ethos (as the historically grown value system of a social group) and the perspective of ethics. Ethical theory and deliberation does not confine itself to a reconstruction of factual norms and attitudes but bears the potential to critically assess moral reasoning and behavior on the basis of principles which claim a more than context-specific validity. Historically grown social norms can be incorporated in ethical deliberation. However, they remain subject to an examination through ethical argument which checks their validity and soundness from a perspective which transcends the specific sociocultural background of their origin. Some ethical theories such as Kantian and Neo-Kantian accounts not only claim a general but a universal validity of their supreme moral principle. This clearly differs from the logic of professional ethos which is bound to a specific field of social interaction, namely the practice of medicine. Universalist approaches to ethical theory are furthermore characterized by the procedural character, i.e. they do not stipulate a concrete course of action as being morally binding under all circumstances but they refer to procedures of moral reasoning, communicative interaction etc.

The critical perspective of ethics is of outmost importance with regard to physicians’ professional ethos if we do not want the medical system to be dominated by the medical professions’ self-understanding alone. Physicians have been in an elitist and powerful position for centuries due to their exclusive knowledge and their monopoly on services which are of utmost importance for all members of society. The professional ethos of physicians can thus be incorporated in ethical evaluation, but must not be the decisive factor regarding topics which reach far beyond physicians’ professional expertise. In the concluding sections of this article the impact of physicians’ professional ethos on the ethical evaluation of morally contentious topics will be illustrated using the example of the current regulation on suicide assistance in German professional law.

### The example of physician-assisted suicide

The question whether a person is morally allowed to end his or her life deliberately has been a subject of philosophical and theological debates since antiquity. Interestingly, in theorizing no clear tendency towards liberalization can be observed, but, over centuries, proponents of suicide alternate with those who regard it as clearly forbidden. The reasons given for the respective positions are manifold and originate in secular as well as religious contexts [28]. The broad discourse however shows that the question about the moral permissibility of suicide is not primarily a medical topic, but that it is essentially linked to the cultural and existential dimensions of human self-understanding [29]. Regarding PAS such a holistic understanding of suicide entails that even if PAS takes place in the physician-patient-encounter there are a number of aspects which are not fully covered by medical expertise. This holds true, for example, with regard the religious and spiritual aspects of suicide or the societal implications of doctor’s assisting their patients to end their life. The medical profession’s occupation with suicides and suicidality, thus, represents only one facet of an empirically complex and multilayered phenomenon. However, medical organizations internationally tend to adopt and advocate positions on this ethically controversial topic ([27], p. 1). In Germany, the impunity of suicide in penal law has a long tradition and expresses the legislator’s explicit intention to refrain from forcing individuals to continue their life and from punishing those who are already in a desperate situation [30]. Following the legal doctrine this means that also the assistance in suicide is not punished by state law. Only suicide assistance in a businesslike form (*geschäftsmäßige Suizidbeihilfe*) is penalized, subsequent to a decision in the German Bundestag in November 2015.

Despite the general non-punishability of suicide assistance by the state, German medical organizations ban this practice in their own professional jurisdiction. The medical professional law (*Ärztliches Standesrecht*) constitutes a specific legal framework which regulates certain aspects of professional conduct and can be regarded as an instantiation of the medical profession’s right to self-legislation. In June 2011, the general assembly (*Deutscher Ärztetag*) of the German Medical Association (*Bundesärztekammer*) decided to prohibit suicide assistance by physicians in the professional framework law (*Musterberufsordnung*), which is a non-obligatory model for the codes of professional law of each individual Federal Chamber of Physicians (*Landesärztekammer*). Up to today, ten of the 17 Federal Chambers of Physicians have adopted this ban in their professional law (*Berufsordnung*), which is binding for all physicians practicing in the respective territories [30]. The remaining seven Federal Chambers of Physicians have not included a passage on suicide assistance at all (e.g. in Berlin) or have developed alternative regulations (e.g. in Westphalia-Lippe).

This recent development not only leads to a regulatory fragmentation in Germany, but also to a situation where a certain type of action which is not clearly penalized by state law is, however, banned by medical professional law. By taking up the topic of suicide assistance, the German medical profession is making a value judgment which reaches beyond the interests of the profession alone: It has an impact on each patient who suffers from a terminal illness and the fear of losing personality and autonomy. If we now recall that the professions’ right to self-regulation is based on the exercise of medical expertise (which is not the same as moral expertise or public decision-making), the broadening of the scope of professional law which takes place becomes questionable.

One could now object that the topic of suicide assistance is only regulated here with regard to *physicians*’ professional conduct and that the professional law does not intend to regulate suicide in general terms. With respect to this objection, we should consider that a profession is distinguished from a mere occupation by the great importance of the professional services for society. It follows, therefore, rather naturally that medical practice often touches on existential topics which potentially affect each citizen in an important way. Examples, such as abortion or euthanasia, show that there are a number of practices related to physicians’ professional conduct which are the main subject of state law (mainly penal law) and cannot be ruled independently by the self-regulation of physicians. These important and morally laden questions about the beginning and the end of life should not be left to the professional ethos of physicians alone.

As a second potential defense of the ban of PAS in professional law, it could be invoked whether physicians’ right to conscientious objection [31, 32] is violated if their professional organizations are no longer in the position to regulate practices such as suicide assistance by themselves. However, this argument is also not very convincing: The impunity of suicide assistance in penal law in no way constitutes a duty for the individual physician to take part in this practice if he or she is asked to do so by a patient. The physician’s evaluation of the unique situation, the application of best medical standards (including comprehensive palliative care) and the physician’s right to refrain from suicide assistance still remain valid and highly important.

One last objection results from the key function of professional ethos to maintain the trust of patients and society. Along these lines the ban of PAS in German professional law could be understood as an expression of the right to regulate the PAS as far as it affects the trust in the profession. However, clear empirical evidence that a liberalization of PAS and other practices at the end of life effectively diminishes the trust in the profession is missing [33, 34] so that the German regulation would have been built on wrong premises. The ban of suicide assistance by professional law, therefore, oversteps the limits of physicians’ professional self-regulation with regard to a question that should not be left to the exclusive legislation of one social group alone.

## Conclusions

The prohibition of suicide assistance in German professional law exemplifies that a distinction between professionalism and ethics in medicine is of more than mere academic interest. Instead, a brief look in the sociology of the profession shows that, next to its undoubted merits, the idea of professionalism is also fraught with the danger of elitism and an exclusive monopoly even on topics which reach beyond the scope of medical expertise. A critical eye must, therefore, be kept on the narrowing of ethics and professionalism in public discussions and in medical education. Morally contentious topics in modern societies should be open to a participatory and inclusive discussion which is not dominated by traditional elites, but is particularly focused on the voices of those who have often been overlooked in the past.

## Abbreviations

PAS, physician-assisted suicide.
